# Parent Experiences in the NICU and Transition to Home

**DOI:** 10.3390/ijerph20116050

**Published:** 2023-06-04

**Authors:** Christine M. Spence, Corri L. Stuyvenberg, Audrey E. Kane, Jennifer Burnsed, Stacey C. Dusing

**Affiliations:** 1 Department of Counseling and Special Education, Virginia Commonwealth University, Richmond, VA 23284, USA; 2 Rehabilitation Science Graduate Program, Medical School, University of Minnesota, Minneapolis, MN 55455, USA; stuyv006@umn.edu; 3 Department of Occupational Therapy, Virginia Commonwealth University, Richmond, VA 23298, USA; kaneae@vcu.edu; 4 Department of Pediatrics, University of Virginia, Charlottesville, VA 22903, USA; jcw5b@virginia.edu; 5 Division of Biokinesiology and Physical Therapy, University of Southern California, Los Angeles, CA 90033, USA; stacey.dusing@pt.usc.edu

**Keywords:** NICU, parent, premature infant, discharge, transition, COVID-19, patient experience

## Abstract

Families (*n* = 12) with infants born at <29 weeks gestation shared their experiences while in the NICU and transitioning home. Parents were interviewed 6–8 weeks after NICU discharge, including some during the acute phase of the COVID-19 pandemic. Findings regarding the parent experience in the NICU were focused around challenges navigating parent-infant separation, social isolation, communication difficulties, limited knowledge of preterm infants, mental health challenges. Parents also discussed supports that were present and supports they wished were present, as well as the impact of COVID-19 on their experiences. In the transition to home, primary experiences included the sudden nature of the transition, anxiety around discharge preparation, and the loss of the support from nursing staff. During the first few weeks at home, parents expressed joy and anxiety, particularly around feeding. The COVID-19 pandemic limited emotional, informational, and physical support to parents and resulted in limited mutual support from other parents of infants in the NICU. Parents of preterm infants in the NICU present with multiple stressors, rendering attending to parental mental health crucial. NICU staff need to address logistical barriers and familial priorities impacting communication and parent-infant bonding. Providing multiple opportunities for communication, participating in caretaking activities, and meeting other families can be important sources of support and knowledge for parents of very preterm infants.

## 1. Introduction

In 2022, 10.5% of infants born in the United States were born prior to 37 weeks gestation [1]. While developmental outcomes for infants born very preterm (<33 weeks gestation) have improved [2], preterm infants remain at significant risk of life-threatening medical challenges and developmental delays [3]. Many parents in the neonatal intensive care unit (NICU) consider interaction with their preterm infant a sign of infant health and vitality [4]. However, during the first 30 days following preterm birth, 80% of infants born at >27 weeks gestation need to spend the majority of their time in an incubator [5]. At term corrected age, the challenges with parent-infant interaction continue as preterm infants are awake ~3% of the day, compared to the term-born infant who is awake 15% of the day [6]. When preterm infants are awake, they tend to fatigue more quickly and provide their parents with more negative and confusing social cues [4,7].

A parent’s sensitive presence in the NICU may buffer neonatal brain dysmaturation and facilitate positive infant development [8,9]. Parents’ abilities to provide sensitive care in the NICU are closely related to their past experiences with parenting and the quality of their support during their infant’s time in the NICU. NICU staff are encouraged to provide family-centered care and value parents as critical members of the NICU team [10]. Family-centered rounds include discussion with family members present, although there is variability in the specific aspects of implementation [11]. Due to the importance of parents being present in the NICU, their mental and physical health during this challenging time has been increasingly explored [12,13]. Despite the establishment of parent involvement as best practice, the technical, fast-paced, and demanding environment of the NICU can be inconsistent in supporting the establishment of parent-provider working relationships [13,14].

Parent-provider relationship challenges in the NICU cause conflicts which may limit parent confidence in their ability to care for their infant while simultaneously contributing to mounting parent stress [15]. Parent stress may also arise from their own medical challenges. Many mothers experienced a personal medical emergency that precipitated the preterm birth of their child and contributed to postpartum mental health challenges [16]. Parent stress and limited self-efficacy may also be related to separation from their preterm infants for extended periods of time [17]. In addition to medical acuity, parents may be separated from infants due to living far away from the NICU, needing to care for their other children, or having to return to work [18]. This separation poses challenges to parent-infant bonding [17] along with increasing parent anxiety, depression, and post-traumatic stress disorder [19]. Compared to the experience of parents with healthy term infants, parents of preterm infants noted elevated stress and negative feelings along with decreased perceptions of social support [20].

Preparation for leaving the NICU contributes to anxiety for parents [21]. Many parents report feeling unprepared for hospital discharge [22]. Despite the risks for more poor developmental outcomes for preterm infants, parents reported that they did not receive long-term developmental information regarding their preterm infant while in the NICU and desired contact information for follow-up, which was not provided in their discharge plan [23]. Financially, families with private insurance struggled with high co-pays related to the multiple NICU discharge follow-up appointments [24]. Many families took a prolonged leave of absence from work, which was required to meet the intensity of their preterm infant’s needs [24,25]. An often-obligatory transition back to work caused many families to struggle with finding adequate childcare. Supports for families at NICU discharge, in best practice, should be readily accessible to families and tailored toward the preterm infant’s unique medical, developmental, and emotional needs. This care should be combined with multidisciplinary early intervention resources and linked to community support [26].

The COVID-19 pandemic contributed to decreased parent support and cumulative stress for families in the NICU [27,28]. During the first year of the COVID-19 pandemic, maternal anxiety was estimated to have increased from 25% to 37% and maternal depression increased from 18–25% to 31% among mothers of newborns [29,30,31]. These percentages are likely to be higher [29] and persist longer among families with infants in the NICU [32]. Stressors for families were also related to loss of childcare, curbed parent-to-parent interactions, decreased social worker availability, and changes in family-centered, multidisciplinary rounds [33,34,35,36]. Some hospitals were able to move to virtual rounds, while others limited the individuals participating [37,38].

This paper describes a sub-study of a larger clinical trial involving intervention support for NICU graduates and their families. The Efficacy of Motor and Cognitive Intervention for Infants Born Very Preterm (SPEEDI-2; NCT03518736) study seeks to determine the optimal timing of intervention delivery within a program that initiates parent education in the NICU and follows the family to support them in their home environment [39]. Parent education is focused on motor and cognitive skill acquisition to support the development of very preterm infants. This phenomenological study [40,41] seeks to understand the parent’s experience while their child was in the NICU and the parent’s experience in navigating the transition from the NICU to home. Due to the historical timing of our interviews, this study is innovative in its exploration of the impact of the COVID-19 pandemic on the parents’ experiences.

### Research Questions

(1) How do parents describe their experience while their preterm child was in the NICU?
  (1a) How did COVID-19 impact parents’ experiences while their child was in the NICU?
(2) How do parents describe their transition from NICU to home?
  (2a) How did COVID-19 impact parents’ transition from NICU to home?

## 2. Methods

The main study and the sub-study were approved by the Institutional Review Board at Virginia Commonwealth University.

Sample: Participants were 13 parents of very preterm infants (*n* = 12), born prior to 29 weeks gestation, and who were cared for in a Level III or IV Neonatal Intensive Care Unit at one of three hospitals in Virginia. Two hospitals were in urban areas and also serve rural communities, and one hospital is in a rural area. The mother and father of one infant were interviewed together with their results compressed. The remaining eleven mothers were interviewed independently. Eligibility criteria for the SPEEDI-2 study included parents’ ability to speak English, be the caregiver of the preterm infant born prior to 29 weeks, and live within a 60 mile radius of one of the three hospitals participating in the study. Participants agreed to participate in this sub-study within 8 weeks of their infants’ discharge from the NICU. Demographic data was collected at enrollment in the SPEEDI-2 study. Please see Table 1 for demographic information about the parents and Table 2 for demographic information about the infants whose parents participated in the sub-study. The children were born between 24 and 28 weeks gestation, with an average gestational age of 26.33 weeks (SD = 1.7 weeks). Infants were born at either extremely low (*n* = 10) or very low (*n* = 2) birth weights with a mean birth weight of 876.5 g (SD = 109 g).

Recruitment: Participants who consented to and enrolled in the SPEEDI-2 study were contacted for participation in the *Parent Perspectives Study* by one of the research coordinators who was not in the role of interventionist or assessor in the main study. Purposive recruitment was used, as all potential participants between were offered the opportunity to participate in the sub-study regardless of group assignment in the main study. Recruitment took place while the infant was still in the NICU or within 8 weeks of discharge, but after group assignment. The research coordinator shared contact information with the interviewer (first author) for potential participants who indicated interest in the sub-study. The interviewer contacted potential participants up to three times to schedule a time to discuss the sub-study and schedule a meeting time. Of the 46 eligible families, 12 (26%) consented to participate in the sub-study. Four infants had been assigned to the SPEEDI_Early group, three infants were in the SPEEDI_Late group, and five infants were in the Control group. At the time of the interviews, only SPEEDI_Early group members had received intervention in the main study. However, the intervention is not addressed in this manuscript.

Measure: The researcher-developed interview protocol was semi-structured, to allow for a range of experiences, feelings, opinions, and knowledge [42] while ensuring that all topics were covered across all interviews. The protocol was reviewed by the study PI and members of the research team, and was piloted with two parents who participated in the SPEEDI intervention but were not eligible for this sub-study. After feedback and piloting, the protocol was revised for clarity. The interview protocol (see Appendix A) included questions designed to elicit information from caregivers regarding their feelings and experiences while their infant was in the NICU and in transitioning home from the NICU, knowledge and supports they currently have or would find useful in parenting a very preterm infant, their sense of competence and confidence in caring for their infant, and their ability to initiate community-based services.

Data Collection: After caregivers indicated interest, they were contacted by the interviewer to set up a time for the interview and were provided with the consent for the sub-study. Interviews occurred via video conference call. Participants were able to choose their location during the call and the interviewer was in a room with a closed door. At the beginning of the call, the interviewer shared the purpose of this study, reviewed the consent form, and responded to all questions about the study. Participants used DocuSign to digitally sign consent after all their questions were addressed. The audio recorded portion of the interview ranged from 12–47 min, with an average of 23 min. Participants were given a gift card in recognition of their time and expertise shared during the interview. Audio files were stored on a secure network drive only accessible to the research team.

Data Analysis: The data analysis team consisted of four White, female, early intervention/early childhood specialists from physical therapy, occupational therapy, and special education. Audio recordings of the interviews were transcribed to a written format by research assistants. Transcripts were uploaded to a password-protected Google drive, and analysis was completed using documents and spreadsheets. Identifying information was removed from transcriptions and all members of the data analysis team except the interviewer remained blinded to the identity and intervention group assignment of each participant. Study participants were identified with aliases. Research team members trained in qualitative methodology analyzed transcriptions using collaborative qualitative analysis procedures [43,44]. Emergent and descriptive coding was used to identify important ideas and themes [44].

Analysis occurred in multiple stages. First, two transcripts were coded independently by four members of the research team (including the interviewer). The team met to discuss potential themes and subthemes and developed an initial codebook. Then six additional transcripts were coded by pairs of team members. During this stage, the team continued to develop the codebook iteratively. The four team members then met to discuss the coded segments and arrived at consensus for each segment in each transcript [43,45].

The final codebook was organized in a 3-code scheme. The first code described *who* was the primary actor in the segment (e.g., parent or extended family), the second code described why the action was discussed (e.g., support or logistics), and the third code described additional factors impacting *who* or *why*, such as COVID-19 or unmet needs. All segments were coded with *who* and *why*, and only applicable segments were coded with the third code. All transcripts were then re-coded by two members of the research team utilizing the final codebook and checked for agreement by the interviewer. Segments with differences were discussed until consensus was reached. Then, three members of the research team reviewed the coded segments organized by *who*-*why* across all interviews to ensure cohesiveness and consistency across coded segments [44].

For the sub-study discussed in this manuscript, the *who* code of “parent” is used, with all applicable *why* codes. *Parent* is defined in this sub-study as the primary caretaker(s) in the household with the infant.

Rigor: Quality indicators for qualitative inquiry were followed throughout the study [46,47,48]. Trustworthiness and credibility of the findings were demonstrated through the use of interdisciplinary collaborative work and development of thick, detailed descriptions in reporting findings. Throughout the data analysis process, team members disclosed and discussed biases that arose regarding parent’s experiences, thoughts, or actions. Team members set aside these biases in order to hear the lived experiences of the family. The study was conducted according to ethical principles and demonstrated meaningful coherence [47].

## 3. Results

### 3.1. Experience While in NICU

The experience of having a very preterm infant who requires intensive medical intervention is unlike other parenting experiences. Parents in the study described how they experienced the idea of parenting a medically fragile, premature infant, spending time in the NICU, barriers they encountered, and support that was available.

Several parents discussed their imagined experience of delivering a medically stable, term newborn, or described a point when they went from hoping to continue on with their pregnancy to gaining knowledge and realizing all of the potential challenges with delivering a preterm infant.

You know, to be completely honest, I was just actually wrapping my mind around being pregnant ... I was starting to show. So, I hadn’t really thought too far ahead into what life would look like with a newborn but that’s okay. She came when she came… I didn’t really picture a lot because like I said I was just trying to focus on the stage I was in at that point. I pictured, you know, just a big baby. A bigger baby. Just more time to relax, more time to not be as worried with everything that she does. Look at her development where she is right now versus having to adjust her age. Is she 9 weeks or is she negative 2 weeks. You know, is she all those kinds of things? Just, I kind of pictured it being a little bit easier. (P1)

The experience of giving birth and not being able to see or hold your child right away was discussed as a difficult experience,

Yeah, not being able to have my hands on my child and having a whole lot of people being able to see her and be around her. I couldn’t see her for a very long time… Her dad got to see her when she- when she came out, I didn’t get to see anything, I didn’t see her face, or nothing. Yeah, they took her straight to NICU. That was a lot for me. (P2)

Another participant shared her experience as someone who has older children, and the difference in her newborn being in the NICU:

Well it was obviously my first experience with a sick baby I would say. Both my first two kids who were born, we never had any medical issues…it was a completely different experience obviously., I was still having a baby and that was exciting to me. Of course I knew about some risk factors and I was scared. But definitely I did not expect what I saw when I entered the room, and I totally freaked out and I just wanted to run and forget about all this. (P3)

Bonding and attachment while in the NICU can be different from what is experienced at home with a newborn, “we couldn’t even touch him for two or three weeks, so that’s a whole different thing” (P4). Despite the challenges and stress, the parents expressed the importance of spending time with their infant to begin establishing a relationship,

I feel like we really did get to bond, you know, it would be sad once I had to go back home cause I didn’t want it to get too late, but that was the only thing-I really did feel like I got to bond with her. (P5)

Parents described the competency and helpfulness of the medical staff, however they also discussed wanting to parent their own child, and how it was different because of the NICU environment. For example, a mother shared, “Yes, she was taken care of, but I wanted her to just be with her mom and I’m hoping that’s what she felt like too. Like, “I just wanna be with my mom” (P6).

Many parents also describe limited knowledge related to the special concerns, particularly medical concerns, with delivering a very or extremely preterm infant, “I didn’t know nothin’ about any of that until they really talked to us about her being premature, of how everything will work with her, and all of that. We didn’t really know anything about that until after she was born” (P7). Another parent shared the difference between having knowledge of child development in general and a preterm infant’s development,

Um, I knew, I knew a little bit of things because I’ve been an aunt at a very young age, so I kind of knew like a little bit of things…So it was like the things that I really knew, they still kind of applied but they didn’t. (P8)

When parents were part of discussions with medical professionals about their child, they described challenges with processing all of the information, “Honestly I really can’t remember. It wasn’t a lot of information with her that I can remember, no. A whole lot of it. (P7)

In contrast, other families were able to take in a lot of information during their NICU experience, which was partially due to their ability to be physically present often,

I took a very active role in the NICU and I was present, engaged with the nurses, engaged with the therapists, you know, I was there. I tried to ask questions, just be present. And I think that naturally lent itself for them to educate more if I’m sitting there going “tell me everything, I want to know. (P1)

Additionally, parents expressed the importance of the major stakeholders having access to information equally.

I got a lot of second-hand information. I would speak with the father of my child, he would always have all of this information and I would be there everyday and they wouldn’t tell me the things that they would tell him so that was very frustrating…. My mom also had the code to access information and she would also be doing the same thing so I’m like, well, how come nobody’s mentioning these things to me and I’m here the most…So I didn’t really like that too much. (P2)

Several parents discussed the impact of having a child in the NICU on their own mental health. While some parents discussed their mental health in regards to the trauma of preterm birth and the NICU experience, others described the toll of isolation. A parent described, “it was definitely an emotional roller coaster. (P4). Another parent discussed her current reflections on that time,

I’ve kind of blocked out the NICU experience, I feel like it may be a trauma response, but I don’t think about it very much, and when I do think about it, I think about it in very vague terms, I don’t think about the specifics very much. (P9)

Parents described reaching out but not having access to what was needed, “I asked for help but towards the end it was just like kind of, all me.” (P8) and “there was one point about midway through our NICU journey, I had a breakdown, cause I was so sad and I wanted to talk to someone” (P9).

Some parents described logistical concerns to being able to spend time in the NICU, such as distance to travel, transportation, and family obligations, ”I was visiting maybe 2–3 times per week, but with a 2 year old…Yeah, especially with the dad being the only one driving. So we only go when we have to go. (P10). Parents also described these logistics as barriers, such as lack of access to current information when they were not able to be physically present in the NICU,

We had certain obstacles where we wouldn’t get updated by some NICU nurses or just for the smallest things but like we explained to them, we live so far away you know the little things that happened may not mean much, you know, on a normal circumstance but we live so far away so any information that we can get to keep us- just informed- is appreciated. (P4)

#### 3.1.1. Support Impacting Experience in NICU

Parents described receiving support from medical staff, their partner, extended family, and peer networks. Although parents described the time while their child was in the NICU as stressful, NICU personnel were often brought up as a source of support and information,

I would say that- she had the best nurses that- honestly, I can’t even explain how great they were … Even like, going beyond what was expected of them, always asking—do you need anything, you know just making conversation, making me feel like I’m not alone. (P5)

While many parents described the helpfulness of hospital staff, there were instances where parental needs were not met, “I talked to the social worker. And she was basically useless … she had no real world experience. She wanted to talk in the hallway of the NICU, which is not a very personal place to talk. (P9). Some parents did not take advantage of support, even if offered, and upon reflection recognized would have been beneficial:

Maybe just somebody to talk to, I think they actually do have that available, I just never called, … I just never utilized it, that might’ve been something I should’ve probably done but…talking to a stranger maybe is a little bit harder …maybe if it had come from somebody that I had trusted or … somebody that I had a good relationship with. (P11)

Several parents shared that they were able to seek out the support they needed from professionals they knew prior to the birth of their child, “before I delivered I had a counselor and so after I delivered early, I started seeing the counselor more. (P12).

When asked about support from other families of infants with preterm babies in the NICU, many parents talked about wanting to connect with other parents of preterm infants, but also discussed many barriers, “I don’t know too many parents to go to basically from the NICU” (P3) while others discussed their pre-existing support network, “I’ve had some friends who’ve been in the NICU so they’ve been supporting me. (P1). A parent shared that there was a lack of connection with other parents having similar NICU experiences due to hospital environmental constraints,

Our NICU was not like open space, everyone had their own rooms so mostly I stayed in my room and I had my two...I knew parents, like their faces, but it wasn’t like you could stop there and talk in the corridor. It’s mostly doctors and nurses running here and there. (P3)

Parents also discussed the unique support they perceived they could receive from other parents who have had children in the NICU due to shared experiences,

And they’re a support system for you, just to have somebody to talk to that has been through it. I guess cause it’s like when you’re in it it’s hard cause you try to explain it to other people what you’re going through, and [spouse] and I can’t talk to people, like our friends that had just had babies or have had babies cause it’s totally different. It’s totally different than a normal experience. (P11)

#### 3.1.2. Impacts of COVID-19 on the Parents’ Experience While in the NICU

During the COVID-19 pandemic, many hospitals changed their visitation policies to restrict the number of visitors allowed. Several of the parents in the study delivered during the most acute phase of the pandemic. These parents expressed only having their partner during this difficult time, “Since COVID happened, it was only, my husband was only allowed to come. It was just the parents and so as far as my parents or anybody else, no one was allowed to come.” (P1). Other parents described how positive supports had been lost:

There was one person that actually (spouse’s name) and I did really like …he was an Episcopal priest, [he] would come by and check on (spouse’s name) and I and the girls, but I think you know, due to the Corona virus he wasn’t in there as much towards after. Visitation was limited and things like that. (P11)

For other parents, COVID-19 just added another layer to the unfamiliar feeling of their NICU experience,

Umm, I don’t know, I guess just how *pause* everything *pause* was so quick. I guess cause COVID is here, and you know, things are different. I didn’t really get a chance to meet a doctor, and my birth, was- it was, unexpec- *pause* I didn’t expect for things to go the way that they did. (P2)

### 3.2. Transition from NICU to Home

The transition from NICU to home includes the process of discharge, as well as the first few weeks after discharge. Many parents greatly anticipated their discharge to home. When their infant needed to remain in the NICU past their original due date, parents expressed disappointment and sadness at the missed milestone,

That last month the girls were in there, I know it was kind of tough, emotionally, it was, I was ready for them to come home and it was past their due date I guess that’s when I started to break down a little bit when it was past their due date cause they say that they usually come home around their due dates so I guess that was a little hard for me. (P11)

Many other parents found the idea of leaving the NICU anxiety provoking,

I didn’t know what to expect. I was so excited the day I was able to bring her home but I was anxious too and just like “is she gonna be okay?” And I felt like for me the transition was easy but I also know that I just wanted to do my best because of the experience she had in the NICU. (P6)

The transition also felt abrupt, almost jarring for some parents, particularly after spending long hours with their infant in the NICU. Many expressed not feeling prepared and having short notice that discharge was happening soon,

You kind of have this long journey in the NICU and it’s so long and day by day and boring, and just a little bit of growth, and then its “oh she’s going home tomorrow.” So, it’s kind of that transition, and you’re preparing for that transition always in the back of your mind, but it just comes really fast. (P1)

The discharge process marked a change in responsibility for care. While the infant was now medically stable, there were additional worries that parents had in becoming the primary caregiver,

Cause they go from being taken care of 24/7 to just being at home with you. So it was nerve wracking. But she was fine, I don’t really remember any real issues that came up. It was more, I was just nervous leading up to her discharge. (P9)

For some parents, their fears and emotions impacted their experience, “transitioning home, it was terrifying” (P4). Parents described their emotions at the change in role to primary caretaker,

I was scared because it’s something that’s a totally new experience and the baby that I didn’t know how to raise for the last four months, my nurse was more of a mother to him than I was at this point. So I was worried and didn’t know about the new environment and stuff. (P3)

When a child is medically fragile, and there is a previous experience with child loss, a protective mechanism can be to not prepare for the child to come home,

But, like really, at the end, when it came down to him coming home, it was like uh, “Oh really, like, is he gonna be our baby now?” You know, I was like, “oh wait a minute, wait, he could stay for a little bit longer. I ain’t got nothing ready yet.” (P4)

Parents also discussed the need for supplies at home and the logistics of having to take over the care responsibilities that the hospital had been providing. Recommendations to ease the jarring feeling of transition included having a “trial run” while still in the hospital,

But I would say that just maybe, I don’t know, having a little more prep even though you’re prepping the whole time, the actual thinking through “okay so if I’m actually gonna be mixing formula, I’m gonna need a bottle for that. If I’m gonna be washing I need drying racks. I need a syringe, I don’t have a syringe.” You know, all those things, the logistical, how am I gonna do what they do at my house. Maybe more like a trial run. I know some hospitals do like the rooming-in, we didn’t do that at my hospital. But, that would’ve been nicer cause we were scrambling “okay well they have all the supplies, we don’t have all the supplies. What do we do?” (P1)

For many parents, the support of NICU staff, particularly nurses, allowed them to transition to home with greater peace of mind:

Yes, they [nurses] answered a lot of my questions. I would tell them as time got closer for her to come home how scared I was and they just let me know that they’re here if I need anything and that it’s a great experience and that I can do it. (P5)

#### 3.2.1. First few Weeks at Home

In leaving the NICU, parents experienced a loss in cutting ties with the nurses they had relied upon. They experienced disappointment when they were told it would be better to reach out to their primary doctor, rather than the NICU team with whom they had build trusting relationships, after discharge to home, “They didn’t tell me, don’t call back up here, but it’s like, you know, if I needed something, it was more like to contact a doctor” (P8). Some parents initially struggled in the stress of the transition,

When we got home, we had like a little, you know, a little disagreement. Oh, I don’t know, I think we were just so, like, “oh my goodness- how do I feed the bottle, how do I do it,” you know, just because it’s a whole new environment. We don’t have the nurses, we don’t have the doctors to tell us everything. But I think after a week I was just in the groove of it. (P4)

While many parents described stress and anxiety with the transition from NICU to home for many reasons, they also just expressed gratitude for being able to bring an infant home,

I really just take care of her and protect her and every day with her is amazing with me, for me. It’s just seeing her grow, seeing her develop more, trying to do more than what everybody really figured that she would really do. She came home doing everything that a normal- she came home in 5 months. (P7)

Aspects of feeding their infants garnered the most discussion when parents described the first few weeks at home. Many parents expressed trepidation around the feeding process,

Their digestive systems to be advanced enough now where it’s like okay now we can feed them at the same time and they’re not throwing up their feeds every time. Cause that was a big thing for the girls too when they first came home. They would- that was always scary too because they’d take their feed and they’d throw up and it’s like, okay, did they get enough? And, you know, so that was a little scary there for the first week or so. (P11)

Parents discussed their stress with their child having a feeding tube, as well as their ability to meet their child’s needs,

Really, to me, caring for a newborn is built-in, you to know how to do it and understand how to do it. But for a preemie newborn that’s coming home with a feeding tube, to me, I feel like is more stressful to a regular baby coming home cause you have more to do with them, more that comes along with them. But, at the end of the day, you still, you know, you’re fine with it. That’s how we always been. We just go with the flow. We fine with it. They well taken care of. It’s just, it comes with a lot but it’s worth it. (P7)

While some parents seemed accepting of infant feeding challenges and changing needs regarding feeding, “And when I give her more she’ll like, take it, you know. I know she wasn’t just being like, greedy so I started feeding her a little bit more” (P8), other parents described difficulty understanding reasons for their child’s feeding challenges,

Feeding has been quite a challenge, and growing. So we have been doing different medications and different combinations of food, breast milk, formula, positions, working with speech therapy to get her to not have pain when she eats so she’ll want to eat so she’ll grow. So, that’s been our biggest challenge. But everything is perfect. She’s not on oxygen, she doesn’t have any tubes or lines or monitors or anything. She’s just a perfect little baby who doesn’t want to eat. (P1)

#### 3.2.2. Impacts of COVID-19 on the Transition Process

While many parents experienced isolation in the NICU related to the pandemic, the transition to home was also greatly impacted. Plans that parents had in place for extended family or friends to provide direct hands-on support had to be modified. Parents also had to determine their level of caution related to the COVID-19 virus, and reconcile their desired level of precautions with those of the important people in their lives,

We thought that was hard and now the girls are home and so we have twins and yeah, every three hours, um, waking up every three hours and then on top of the Coronavirus, it’s been quite challenging, cause you can’t really get help from anybody…We thought our moms were going to be more helpful and [spouse’s] mom, she’s scared to leave her apartment and everything like that, and my mom is kind of the opposite, I don’t think taking it as seriously as she should. (P11)

Some parents used technology to connect with others and to get the emotional support they needed. They discussed introducing their infant to extended family members via video,

Havin’ a COVID baby…he really got adjusted to seeing people like we doin’ now, video time, you know. His grandmother, my grandmother, my mom, you know everybody. So he loves the camera and he’ll show off for the camera, he’ll talk, he’ll play, and stuff. (P4)

One parent expressed concern about having to limit their older child to protect their newborn from COVID-19 exposure, and the worry regarding the unknown,

Well, it’s been a little bit confusing, like whether I should send my son to school or not, cause I’m not that worried about my son getting COVID, cause you know it is not that common in 3 year olds to get a very serious form of it. But you know, I’m not really sure how susceptible she is to it. And no one really does. That’s been the hardest, worrying about pathogens. You know preemies are different, but she’s been a good baby so far. She’s sleeping well, once we got the reflux controlled. She really started sleeping nicely at night. (P9)

## 4. Discussion

Through our semi-structured interview process, parents shared their experiences of having a very preterm infant in the NICU and their experiences when transitioning home. While in the NICU, limited knowledge of preterm birth outcomes coupled with lack of control over maternal and infant health crises contributed to feelings of loss and shock for parents shortly after delivery. Knowledge, given the timing of events, has the potential to decrease or increase parent stress [49]. In this study, parents described stress when possessing knowledge of their impending preterm delivery with little control over the situation [50]. Traumatic events are generally hallmarked by experiencing extreme distress along with helplessness in escaping the situation [51]. In a study including 86 mothers of infants in the NICU, 35% presented with acute stress disorder 5 days after birth that transitioned to post-traumatic stress disorder 35 days after preterm delivery for 15% of mothers [52]. In our sample, stress related to the preterm experience appeared particularly salient for families who had limited access to information or support based on logistics or family circumstances.

In addition to increased signs of trauma related to persistent stress, parents of infants requiring a NICU stay also experience increased levels of depression and anxiety [32,53]. Many NICUs have staff whose role is to support family mental health and assist families in accessing mental health resources upon discharge [54]. However, parents in our study discussed the need for more support or support that met their needs at the salient moment in time, provided in a conducive, private environment. While nursing was able to provide emotional support to some families, others were limited in their ability to receive comfort from nursing, social work, or other medical staff. Some parents discussed having emotional support offered to them, but they had not felt ready or available to engage in the types of support offered due to a lack of trust, lack of privacy, or limited bandwidth to engage. Having multiple avenues and timepoints to participate in activities which support mental health may help parents to take advantage of the resources available [18,55].

Parents frequently discussed communication challenges, both with the timing and the format of receiving information. Despite best practices of including parents as a member of the care team [14], few parents discussed communicating with staff as a partner in their infant’s care. Many parents described communication as one-way, in that they were passive receivers of information, rather than actively engaged in two-way discussions. This may not fully reflect their experiences, but rather the salient stories they chose to share. Challenges with medical staff/family communication have been related to parental feelings of not being welcomed in the NICU and parents questioning their abilities to care for their infant at home [23,55,56].

Interviews revealed differences in family expressions regarding their infant’s state of mind, including their infant’s perceptions of being cared for during their NICU stay. In particular, parents who expressed fewer thoughts of their infant’s emotional and arousal states also had a more difficult time with developmentally appropriate representations of their infant’s behaviors. Subsequently, these parents were also more likely to describe their infant as “greedy” or “lazy”. These challenges most often related to feeding prior to NICU discharge [57]. Family challenges with describing their infant’s experience from a sensitive and developmentally-appropriate viewpoint may be indicative of struggles with developing a secure parent-infant relationship [58]. Sensitivity to an infant’s cues has been described as even more critical in the context of parent-pre-term infant relationships than parent-term born infant relationships [59]. Sensitivity and responsiveness within the parent-pre-term infant relationship has been related to positive changes in both brain development [8,60] and developmental outcomes [9,61].

Parents’ emotional and physical separation from their infant during the NICU stay contributed to challenges with the transition to home. Parents in this study expressed loss and grief for the time they did not have with their newborn infant, along with wanting to participate in daily care routines for their infant themselves, such as bathing or feeding. This lack of ability to participate in care routines for the infant contributed to limited parent self-efficacy and less opportunity for bonding [62,63]. When parents lacked self-efficacy, they expressed more anxiety in the transition to home and more feelings of being unprepared for the transition. This parent emotional dysregulation was also expressed in both fear and in perceptions of discharge as hectic or chaotic. When parents in this study felt supported by nursing and felt empowered to ask for training to their comfort level prior to discharge, they subsequently expressed less overall anxiety with leaving the hospital to care for their infant at home.

Overall, the transition from NICU to home was described as frantic and rushed. While parents eagerly awaited the time that their infant could come home, communication related to timelines and expectations was reported to be difficult and confusing. Discharge planning should begin early, and include information about specific developmental and medical outcomes that need to be achieved, as well as what parents need to do to prepare for their infant to come home [55]. Parents described waiting for a far-off date and then suddenly hearing that discharge was happening within the next 48 h, leaving them little time for the physical preparation and challenging logistics of transition a very preterm infant to their home. Having a visual timeline or tangible way to mark milestones may be helpful for parents as they anticipate the future transition process.

The COVID-19 pandemic caused families to struggle with understanding vague and inconsistent lock-down guidelines for NICU infants which posed unique challenges in finding both physical and emotional support [64]. One mother was not able to meet her doctor prior to the unexpected delivery. Multiple families who had infants in the NICU during the most acute phase of the pandemic shared the difficulty with access to physical support and the restrictive visitor policies. While parents understood why the policies were in place, they still perceived a negative experience. Parents also discussed the changing expectations once they were home. Extended family was meeting their child via video conference rather than in person and in-person caregiving support was less than previously planned due to restrictions or differing approaches to pandemic precautions.

### 4.1. Implications for Practice and Policy

The results from this qualitative study yielded many insights and parent perspectives that will be helpful to clinicians engaging with families during their NICU stay or during their transition to home. The NICU staff’s primary role is to provide lifesaving medical care for the infant. However, staff also need to support parents so that parents may then sensitively address their preterm infant’s emotional and developmental needs. Additionally, processing information was difficult for most families when experiencing significant distress and loss of control. However, when families experienced periods of relative stability, they perceived greater control and retained more knowledge. Differences in access to communication existed based on logistical factors such as transportation availability, competing time priorities, caregiving duties, and employment demands. Discussions should occur with each parent, despite their presence in the NICU, regarding their preferred communication method and timing [65].

Transition to home was reported to be stressful and abrupt. While all parents knew, and were excited about, the upcoming discharge, many discussed how jarring it was to be told that discharge was imminent. Some NICUs have rooming-in as part of the discharge process, where the infant is no longer cared for directly by the medical staff and parents have caretaking responsibilities with a nursing safety net available for emergencies. Rooming-in in the NICU has been related to decrease preterm infant distress as measured by salivary cortisol [66]. Implementing this as part of the discharge process, or making it available to those who want the opportunity, may help alleviate some of the anxiety parents reported. Consistently providing parent education related to medical equipment for the home and child development information regarding premature infants, particularly feeding, could also support families during this time of transition [55].

Given the timing of this study during the pandemic, we acknowledge that supports previously in place were temporarily suspended. However, it may be important to consider opportunities for parent-to-parent support while families remain in the NICU to allow parents to connect with other parents of very preterm and NICU grads [18] as the effectiveness of peer-to-peer support after NICU discharge has not been consistently demonstrated [67]. It could be helpful for NICU staff, practitioners, and researchers to develop specific guidance related to family-centered care, both during the infant’s stay in the NICU and the transition period. Having rounds or physician availability during times that families are present in the NICU or available via phone, could increase family-centeredness. A “warm handoff” to community-based medical specialists, therapists, and Part C Early Intervention could support families during this stressful period of time.

### 4.2. Implications for Research

Future research may explore the type and frequency of support that parents would like for themselves while their child is in the NICU. Additionally, more information is needed regarding parents who have experienced the cumulative effects of more than one of their pregnancies resulting in NICU services for their infant. Along these lines, future research could examine the supports that would be beneficial for parents experiencing child loss in the NICU and then delivering subsequent infants requiring NICU care.

### 4.3. Limitations

This study included a group of parents enrolled in a larger intervention study. While we do not know motivations for agreeing to the interviews, these parents may or may not be representative of parents of preterm infants. Parents are recalling a stressful time in their lives and may not have shared as many positive moments, even when prompted. The original design for this study included in person interviews in the parents’ homes. Due to the onset of the COVID-19 pandemic at the beginning of data collection, all interviews were conducted via teleconference. Parents may not have had much familiarity with this technology at the beginning of the pandemic and this gap in comfort may have impacted the information they shared with the researcher. However, the depth of information shared during the interviews mitigate some of the concerns with technology.

## 5. Conclusions

This study affirms previous research in hearing parent’s perspectives on their time in the NICU and adds to the literature by sharing the unique experience that the COVID-19 pandemic had on parent experiences. This study also builds on previous research with parents, given that the participants had a more diverse background than have been included in previous studies. While parents shared that they struggled with many aspects of this time, they also shared their moments of joy.

Parents of preterm infants in the NICU have multiple stressors and attending to parental mental health is crucial. Separation from their infant, not having the primary caregiving role for the first few months of their infant’s life, disjointed communication, limited knowledge regarding development for very preterm infants, minimal emotional support from others who understood parenting a premature infant, and a transition process that was perceived as chaotic and hectic had significant impact on the parents. Families were also impacted by logistical and familial priorities, including multiple caregiving responsibilities, employment, and traveling long distances to the hospital, which impacted communication and ability to spend time in the NICU.

Clinicians are urged to think creatively about how to support parents during this emotionally challenging time. Providing multiple opportunities for communication, participating in caretaking activities, and meeting other families can be achieved through a variety of ways. Celebrating small milestones, utilizing video conferencing, offering multiple types of support, and asking for input from families are minor, but impactful, options that could support parents and their infants. While the acute phase of the pandemic has passed, information learned regarding availability of support and the impact of restricted visitation can inform future work.

## Figures and Tables

**Table 1:** Parent Demographics.

Race		
	Mother (*n* = 12)	Father (*n* = 12)
Asian	1	1
Black/African American	4	6
Native Hawaiian or Pacific Islander	0	0
White	5	4
Biracial	1	0
Not Reported	1	1
Highest Educational Level		
	Mother (*n* = 12)	Father (*n* = 12)
Some High School	1	1
High School/GED	2	3
Some College	1	2
Associate’s degree	0	1
4 year degree	4	1
Graduate degree	3	3
Not reported	1	1

**Table 2:** Child Demographics (*n* = 12).

Birth Weight	
600–699	1
700–799	4
800–899	2
900–999	3
1000–1099	2
Weeks Gestation	
24	1
25	1
26	4
27	5
28	1
Days in NICU	
56–62	1
63–76	1
77–90	1
91–104	3
105–118	1
119–132	3
133–146	0
147–160	1
161–174	0
175	1
Assisted Ventilation	
None	0
1–2 days	0
3–14 days	1
15–28 days	2
29 or more days	9
Periventricular Hemorrhage	
None	9
Grade I or II	1
Grade III or IV	2
Neonatal Medical Index	
1	0
2	0
3	1
4	2
5	9
Necrotizing Enterocolitis	
No	9
Yes	3
Race	
Asian	1
Black/African American	4
Native Hawaiian or Pacific Islander	0
White	4
Biracial	2
Not Reported	1

## Data Availability

At this time, data is not publicly available. Please contact the first author for requests.

## Appendix A

**Interview Protocol**

(1) As you reflect on your journey over the past few months, what stands out the most to you?
(2) What did you know about infant development and parent-infant relationships prior to the birth of your child?
  (a). How did you know this information? Was it related to your educational experience, occupation, previous experience parenting, or other factors?
  (b). What did you envision when you pictured the birth of your child and the first few weeks at home?
(3) What supports did you have as you transitioned home from the NICU?
  (a). Probe if no immediate response: This could include developmental or medical information, contact information, planning for logistics with medical follow-up, transportation, etc.
  (b). What supports would have been useful?
(4) What are your child’s current needs, related to their medical history?
(5) Due to the NICU experience and the specific needs you just described, how do you view your role as mom or dad? What are the important day-to-day events?
(6) Who is currently supporting you and your child based on your child’s specific needs? This could include medical personnel, therapists, community agencies, etc.
  (a). Who comprises your emotional support network? Do you have local support?
  (b). Who comprises your informational support network? Who do you turn to when you have questions about your child?
  (c). Are your material needs met or what needs do you have? For example, do you have access to all the medical equipment needed to support your child, have transportation needs, or has your work schedule/hours changed due to the support needs for your child?
(7) What do you know about early intervention or Part C services?
  (a). Prompt if no immediate response: This is a program for infants and toddlers with or at-risk for disabilities and developmental delays. Your child may qualify to receive services due to their preterm birth.
  (b). Describe any contact you have you had with Early Intervention / Part C. This could include a conversation with a service coordinator prior to discharge from the NICU, assessments, or visits at home with an educator or therapist.
  (c). (When was the first point of contact with EI/Part C?
  (d). Do you have an IFSP developed?
(8) As you think about supporting your child’s growth and development, what sources of information have been useful?
  (a). Probe: These could include conversations with other parents, medical staff, or therapists, reading books, looking at websites, etc.
(9) SPEEDI_Early Participants only: Was the frequency of the visits too much, just right, or not enough? Why?
  (a). You recently completed Phase 1, which was the part of the study in the NICU when you met with the therapist 5 times in 2–3 weeks -
    - How convenient was it to schedule visits while in the NICU?
    - What were the biggest barriers to scheduling visits?
    - How many caregivers participated directly in SPEEDI visits?
    - As a caregiver that was present during SPEEDI visits, did you feel capable to share information with others (i.e., non-attending parent, grandparent)? What would have been beneficial for you in sharing the information?
    - Were you able to implement what you learned from the therapist into your daily interaction with you child immediately? Or did you feel like it only worked during therapy or it took time for the work to pay off?
(10) What are your favorite activities to do with your child?
(11) What concerns do you have about your child’s development?
(12) Do you have any concerns about your ability to meet your child’s needs?
  (a). Do you have any concerns about your ability to bond and care for your child?
(13) Are there any other aspects of the transition home from the NICU or parenting during these first few months that I should know?
  (a). Probe: You may want to reflect on if parenting was different than you expected it to be, in regards to the medical component as well as your ability to socialize with friends/family, view yourself as a mom/dad/caregiver, support your other children, work, engage in previous routines (exercise, etc.)
